# Preliminary Field Evaluation of a Low-Cost IoT Workflow for Dissolved Oxygen Monitoring and Short-Horizon Forecasting in Nile Tilapia Aquaculture

**DOI:** 10.3390/s26134242

**Published:** 2026-07-04

**Authors:** Ahmed Mohammed Al-Khaldi, Ragavesh Dhandapani, Mohammed Ahmed Al-Badri

**Affiliations:** Department of Electrical and Communication Engineering, College of Engineering, National University of Science and Technology, P.O. Box 2322, Seeb 111, Oman; ahmed200251@nu.edu.om (A.M.A.-K.); mohammed200376@nu.edu.om (M.A.A.-B.)

**Keywords:** Nile tilapia, *Oreochromis niloticus*, dissolved oxygen, low-cost IoT, preliminary field evaluation, aquaculture monitoring, short-horizon forecasting

## Abstract

Short-term fluctuations in dissolved oxygen are difficult to capture in warm outdoor Nile tilapia (*Oreochromis niloticus*) ponds using periodic manual measurements, yet they strongly influence fish performance and farm management. This study presents a preliminary field evaluation of a low-cost IoT workflow for dissolved oxygen monitoring and short-horizon forecasting in pond-based tilapia culture. An ESP32-based sensing node continuously measured dissolved oxygen, temperature, and pH, transmitted readings to a cloud backend, and generated short-horizon forecasts from 5 min aggregated windows. During live validation from 1 to 10 April 2026, the 30 min forecast achieved a mean absolute error of 0.783 mg/L and directional accuracy of 60.23%, with only modest improvement over a persistence baseline. The 6 h forecast achieved 1.109 mg/L and 53.82%, respectively, indicating limited predictive value at the extended horizon. An extended 47-day field deployment (May–June 2026) captured four sensor-recorded low-DO events and two documented power outages, causing sensor downtime and providing additional field-deployment evidence. These results demonstrate the engineering feasibility of the integrated workflow, but they do not establish robust operational forecasting validity because the data were collected from one pond, high-frequency records were temporally correlated, and independent reference-meter validation was not available. The study is, therefore, best interpreted as a proof-of-concept field evaluation that identifies practical requirements for future low-cost aquaculture forecasting systems.

## 1. Introduction

Aquaculture now supplies a larger share of global aquatic animal production than capture fisheries, and its continued expansion is central to future aquatic-food security [1]. As culture systems intensify, reliable water-quality management becomes increasingly important because production losses are often driven by short-term excursions in key environmental variables rather than by slowly changing average conditions alone [1].

In Oman, aquaculture is acquiring greater strategic importance within broader efforts to strengthen food security, diversify production, and expand blue-economy activity [2]. The sector includes both marine and freshwater farming, and Nile tilapia (*Oreochromis niloticus*) is now popularly farmed in several parts of the country [3]. Nile tilapia is also one of the most important cultured freshwater fish globally because of its relative hardiness, broad environmental tolerance, and suitability across a wide range of farming systems [4]. FAO-based national production summaries further indicate that Nile tilapia contributed 22.5% of Oman’s aquaculture production quantity in 2018, which reflects its local relevance alongside its wider aquaculture importance [5].

For Nile tilapia culture, dissolved oxygen (DO) is one of the most operationally important water-quality variables because oxygen availability directly affects feeding activity, metabolism, feed conversion, and growth performance [6,7]. Research has shown that DO concentration significantly influences feed intake and growth in tilapia: fish below 100 g show reduced feeding when DO falls below 3 mg/L, while larger fish (above 200 g) continue to increase feed intake as DO rises from 2.6 to 6.0 mg/L, suggesting that larger fish have higher oxygen requirements for optimal performance [7]. Low DO, therefore, matters not only during acute mortality events, but also during sublethal episodes that reduce appetite and productivity before overt distress becomes visible [6,8]. In warm-water pond systems, DO is shaped by interacting biological and operational drivers, including phytoplankton photosynthesis, fish and microbial respiration, feeding, organic loading, aeration, and water exchange [9]. Tropical earthen ponds are characterised by daily DO fluctuations, with nocturnal respiration often leading to hypoxia that persists for several hours [8]. As a result, periodic manual measurements may fail to detect short-duration deterioration early enough for timely intervention [9].

These constraints have driven growing interest in Internet of Things (IoT)-based aquaculture monitoring, in which water-quality sensors, wireless communication, and cloud or edge analytics are used for continuous observation and remote visibility [10,11]. Recent reviews show that temperature, pH, and DO remain among the most frequently monitored variables in smart aquaculture, and that IoT-enabled monitoring can improve management responsiveness and reduce production risks [10]. However, monitoring alone addresses only part of the operational challenge: continuous sensing provides real-time visibility, but operators still require advance notice to prepare interventions before critical thresholds are reached. Furthermore, practical IoT deployment remains challenging. Aquaculture IoT systems must contend with sensor maintenance, calibration drift, fouling, unstable power or communication, and the uneven reliability of low-cost sensors under real pond conditions [11,12]. These monitoring limitations are particularly relevant when systems are intended for smaller-scale or cost-sensitive farms, where affordability is important but technical fragility can quickly weaken practical value [12].

In parallel, DO forecasting has become an increasingly important component of intelligent aquaculture management. Recent reviews show that predictive approaches now span statistical models, ensemble methods, deep learning, and hybrid frameworks, with the broader goal of enabling more proactive intervention and more efficient resource use [13]. Earlier work in aquaculture had already identified short-term DO forecasting as a potential basis for early-warning support [14]. More recent studies have moved closer to integrated monitoring and prediction in live aquaculture contexts, including low-cost edge deployment in tilapia systems and IoT-based predictive modelling in tropical tilapia ponds [15,16]. Taken together, however, this literature still suggests that comparatively less attention has been given to field-evaluated workflows that connect low-cost sensing, cloud-side aggregation, feature engineering, operational event logging, dashboard-based monitoring, and delayed validation against subsequently observed measurements under real pond conditions [10,11,12,13,14,15,16].

This gap between offline model development and operational deployment is important from a farm-management perspective. For pond operators, the key question is rarely whether a model performs well on a historical benchmark alone; it is whether a low-cost system can produce forecasts that can be evaluated under operational conditions despite practical constraints in sensing, communication, and maintenance. Live pond evaluation is relevant because it exposes models to real diel oxygen cycles, biological variability, and operational disturbances that laboratory or tank-based evaluations may not capture. This issue is particularly relevant for Omani tilapia culture, where freshwater aquaculture is developing, Nile tilapia is locally important, and short-horizon DO management is a practical operational need rather than a purely methodological exercise [2,3,5].

Accordingly, this study presents a preliminary field evaluation of a low-cost IoT workflow for dissolved oxygen monitoring and short-horizon forecasting in Nile tilapia pond aquaculture. The objective is not to introduce a new forecasting algorithm or to claim a generalisable operational warning system. Instead, the study evaluates how a low-cost sensing-to-forecasting workflow behaves under outdoor pond conditions, including cloud data handling, operational event logging, stored forecast matching, sensor instability, power interruptions, and limited threshold-event evidence. By reporting both performance metrics and practical limitations, the study provides a proof-of-concept assessment intended to guide future development of longer-term, multi-pond, independently reference-validated aquaculture forecasting systems.

## 2. Materials and Methods

### 2.1. Study Setting and Pond Deployment

This study monitored freshwater Nile tilapia (*Oreochromis niloticus*) at a family-operated tilapia farm in North Al Sharqiyah Governorate, Oman. The deployment site consisted of an outdoor concrete pond with a water volume of approximately 12 m^3^ (12,000 L) and a depth of approximately 1 m. The pond was stocked with approximately 100 Nile tilapia supplied with groundwater. Fish weight measurements recorded during the study period indicated average weights ranging from 36 g to 111 g, corresponding to fingerling to early-mature growth stages based on standard tilapia weight classifications [7]. The pond operated under typical small-scale aquaculture conditions, with daily feeding, weekly partial water exchange, and continuous aeration using an air pump with diffusers.

**Figure 1:**
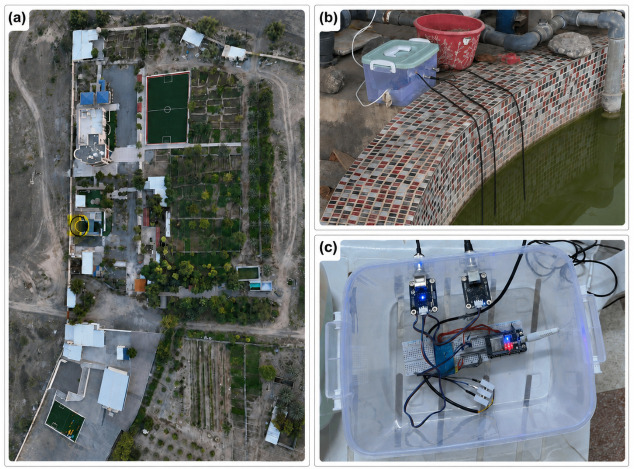
Pond deployment and monitoring hardware. (**a**) Aerial overview of the family-operated tilapia farm in North Al Sharqiyah, Oman. (**b**) Sensor placement at the outdoor concrete pond, with DO, temperature, and pH probes positioned below the water surface. (**c**) ESP32-based monitoring node showing the microcontroller, sensor interface boards, enclosure, and sensor connections.

Sensors were positioned in the upper water column, approximately 20 cm below the water surface (corresponding to 20% of pond depth), consistent with recommended sampling depths for water quality monitoring in small-scale aquaculture ponds [17]. The monitoring system was deployed at the pond site and operated continuously during the live validation period (1–10 April 2026). During initial deployment, practical challenges were encountered, including electrical instability and analogue signal noise, consistent with previous reports of calibration drift, placement sensitivity, and field instability in low-cost aquaculture monitoring [11,18]. These issues were addressed through improved power regulation, cable shielding, and sensor positioning adjustments. After these adjustments, the final configuration operated throughout the validation period with reduced anomaly rates (Figure 1).

### 2.2. Monitoring Platform and Sensor Configuration

Water quality was measured using an ESP32 DevKit V1 microcontroller (Espressif Systems, Shanghai, China) [19] connected to three sensors: a galvanic dissolved oxygen probe (SEN0237-A, DFRobot, Shanghai, China) with signal-conditioning board [20], a waterproof DS18B20 digital temperature sensor (Maxim Integrated, San Jose, CA, USA), and a Gravity Analog pH Sensor Kit V2 (DFRobot, Shanghai, China) [21] (Table 1). Sensors were positioned below the water surface within a protective in-water casing to ensure consistent submersion and protection from direct sunlight [22]. The pond aeration system (air pump with diffusers) maintained suitable dissolved oxygen levels for the stocked tilapia. Electronics were housed in a protective waterproof enclosure positioned at the pond edge.

The sensing-node hardware cost was approximately 112 OMR, including the ESP32 microcontroller, DS18B20 temperature sensor, Gravity pH Sensor Kit V2, galvanic dissolved oxygen sensor, and protective enclosure. The largest cost components were the dissolved oxygen sensor (68 OMR) and pH sensor (28 OMR), while the ESP32 microcontroller and temperature sensor cost OMR 7 and OMR 4, respectively.

**Figure 2:**
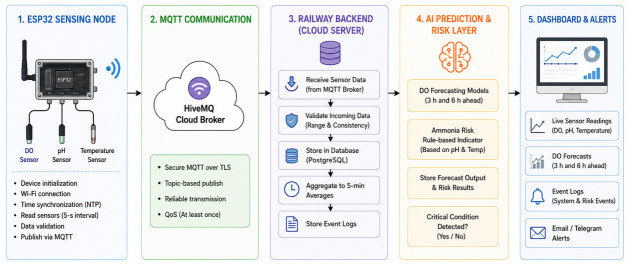
Monitoring platform showing sensing node, MQTT communication, cloud backend, prediction layer, and dashboard.

Sensor readings were transmitted via MQTT to a HiveMQ Cloud broker using topic-based routing. A Python V3.10 backend hosted on Railway received incoming readings, performed validation, stored raw records, generated aggregated windows, prepared prediction features, triggered forecasting, and logged outputs. Persistent CSV-based storage retained raw readings, aggregated records, forecasts, validation logs, and event logs. A web dashboard displayed live readings, historical trends, forecasts, alerts, and a rule-based ammonia-risk indicator. The system was designed for operator-facing monitoring and trend-awareness information rather than autonomous control (Figure 2).

### 2.3. Sensor Calibration and Measurement Quality Control

All sensors were calibrated before deployment to reduce measurement bias [12]. The DS18B20 temperature sensor was calibrated using a three-point procedure (cold, room-temperature, and heated water) against a reference digital thermometer; linear regression yielded the temperature calibration equation (Equation (1))

(1) $$T_{\mathrm{corrected}}=1.0877\cdot T_{\mathrm{raw}}-1.3862$$

with a post-calibration deviation of ±0.3 °C. The dissolved oxygen probe was calibrated using two-point scaling between zero-oxygen (chemically deoxygenated using sodium sulfite) and air-saturated water [23]; the final conversion is given by Equation (2)

(2) DO=13.648·V

where *V* is probe voltage, with verification showing 8.3–8.6 mg/L in aerated water at ambient temperature. Dissolved oxygen saturation is temperature-dependent, and galvanic DO probe output can be affected by thermal conditions [23]. In this prototype, DO calibration was performed using air-saturated water at the ambient deployment temperature, and the resulting conversion was verified under similar pond-temperature conditions. Temperature was monitored continuously using the DS18B20 sensor; however, dynamic temperature compensation was not implemented in the real-time DO conversion. Therefore, the calibrated DO values should be interpreted as field-verified values under the observed deployment conditions rather than as fully temperature-compensated measurements across a wide thermal range. The pH sensor was calibrated using certified pH 7.00 and pH 4.00 buffer solutions [24], yielding Equation (3)

(3) pH=−5.839·V+15.164

with a deviation of ±0.05 pH units. pH readings showed greater sensitivity to drift and were, therefore, retained as a supporting variable rather than for critical alerting (Table 2).

Calibration was conducted before deployment and was not repeated during the 10-day live validation period. For longer-term deployments, periodic recalibration would be advisable to account for sensor drift [12]. Additionally, galvanic dissolved oxygen probes and pH electrodes require regular cleaning to prevent biofouling and membrane degradation, which can affect measurement accuracy in pond environments [25]. During the deployment period, sensors were visually inspected, but formal cleaning protocols were not implemented due to the relatively short monitoring duration.

### 2.4. Data Acquisition, Aggregation, and Storage

The ESP32 sampled and calibrated dissolved oxygen, temperature, and pH at 5 s intervals and transmitted readings via MQTT to the cloud backend. Timestamps were synchronised using Network Time Protocol (NTP) on the ESP32 at startup, ensuring consistent time alignment between sensor readings and server-side processing. Incoming readings were validated, stored as raw records, and used to update the live dashboard. Before prediction, raw readings were aggregated into 5 min windows (nominally 60 readings per window). For each window, summary statistics were computed: mean, minimum, maximum, standard deviation, and change from the previous window. This aggregation reduced sensor noise and produced a stable time-series representation suitable for forecasting [26]. Windows with insufficient readings due to temporary communication interruptions were flagged and excluded from forecasting to avoid unreliable predictions. Operational events (feeding and water changes) were logged manually with timestamps via the dashboard interface.

Data were organised into two phases (Table 3). The offline model-development dataset (March 2026) supported training, validation, and test-based model selection with 2034 total samples. The live pond deployment dataset (1–10 April 2026) contained 102,670 raw readings and 3041 aggregated windows collected from the operational tilapia pond, enabling validation by comparing stored forecasts against subsequently observed dissolved oxygen values.

### 2.5. Feature Construction and Prediction Dataset Preparation

Features were constructed from 5 min aggregated windows to represent current state and recent temporal behaviour. Four feature categories were used, each selected based on known drivers of dissolved oxygen dynamics in aquaculture ponds [9,13]. First, statistical features (mean, minimum, maximum, standard deviation, and change) for dissolved oxygen, temperature, and pH captured current water-quality state and short-term variability. Temperature was included because oxygen solubility decreases with increasing water temperature, and pH reflects biological activity that affects oxygen balance [9]. Second, lag features (previous window values and rolling statistics over 30 min and 1 h windows) captured recent dissolved oxygen trajectory, which is relevant because short-term DO changes often follow persistent patterns driven by respiration and photosynthesis cycles [8]. Third, time-of-day features (hour encoded cyclically using sine and cosine transformations) represented diurnal patterns without introducing discontinuity at midnight, reflecting the well-documented diel oxygen cycle in warm-water ponds where photosynthesis dominates during daylight and respiration causes nocturnal oxygen decline [8,9]. Fourth, event features (time since feeding and time since water change) captured gradual operational effects, as feeding events increase biological oxygen demand through fish metabolism and uneaten feed decomposition [6].

Prediction targets were prepared separately for each horizon: 30 min ahead (6 windows) and 6 h ahead (72 windows). Rows without valid future targets were excluded from training and validation. Quality checks ensured forecasting occurred only when sufficient recent data were available (Table 4).

### 2.6. Forecasting Models and Offline Temporal Evaluation

The prediction task was formulated as supervised regression with two forecast horizons: 30 min (primary trend-awareness output) and 6 h (extended planning indicator). Candidate models included Linear Regression, Ridge Regression, Support Vector Regression, Random Forest [27], XGBoost, LightGBM [28], and ensemble averaging (Table 5).

Model development used chronological train–validation–test splitting to prevent temporal data leakage. The offline dataset contained 2034 samples: 1525 for training, 305 for validation, and 204 for testing. Tree-based ensemble methods (Random Forest, XGBoost, and LightGBM) were prioritised because they handle non-linear relationships in tabular time-series data effectively and have shown strong performance in aquaculture water-quality prediction [13]. Linear models (Linear Regression and Ridge Regression) were included as baselines but showed higher errors due to the non-linear nature of dissolved oxygen dynamics.

Hyperparameter tuning was conducted using randomised search (50 iterations per model) with internal cross-validation on the training set, followed by final model selection based on the held-out chronological validation set. For Random Forest, tuned parameters included number of estimators (50–300), maximum depth (5–30), minimum samples split (2–10), and minimum samples leaf (1–4). For LightGBM, tuned parameters included number of leaves (20–150), learning rate (0.01–0.3), number of estimators (50–300), and regularisation parameters. Performance was evaluated using mean absolute error (MAE), root mean squared error (RMSE), coefficient of determination ($R^{2}$), and directional accuracy. Based on validation performance, Random Forest was selected for the 30 min forecast because it achieved the highest $R^{2}$ and directional accuracy with validation MAE close to the best-performing candidates, while LightGBM was selected for the 6 h forecast because it achieved the lowest validation MAE and highest $R^{2}$ among the evaluated models.

### 2.7. Live Cloud Deployment, Validation, and Recorded Outputs

After offline model selection, the selected models were integrated into the Railway-hosted backend for operational evaluation at the tilapia pond. This live pond deployment (1–10 April 2026) exposed the forecasting system to real aquaculture conditions, including natural diel oxygen cycles, fish respiration, feeding events, and environmental variability, which laboratory or tank-based evaluations may not fully capture. During deployment, the system generated forecasts continuously, logged each prediction with its timestamp and input features, and later compared stored forecasts against actual dissolved oxygen values observed after each horizon elapsed. Validation matching used a 2.5 min tolerance window to account for minor timing variations between forecast timestamps and observation timestamps.

**Figure 3:**
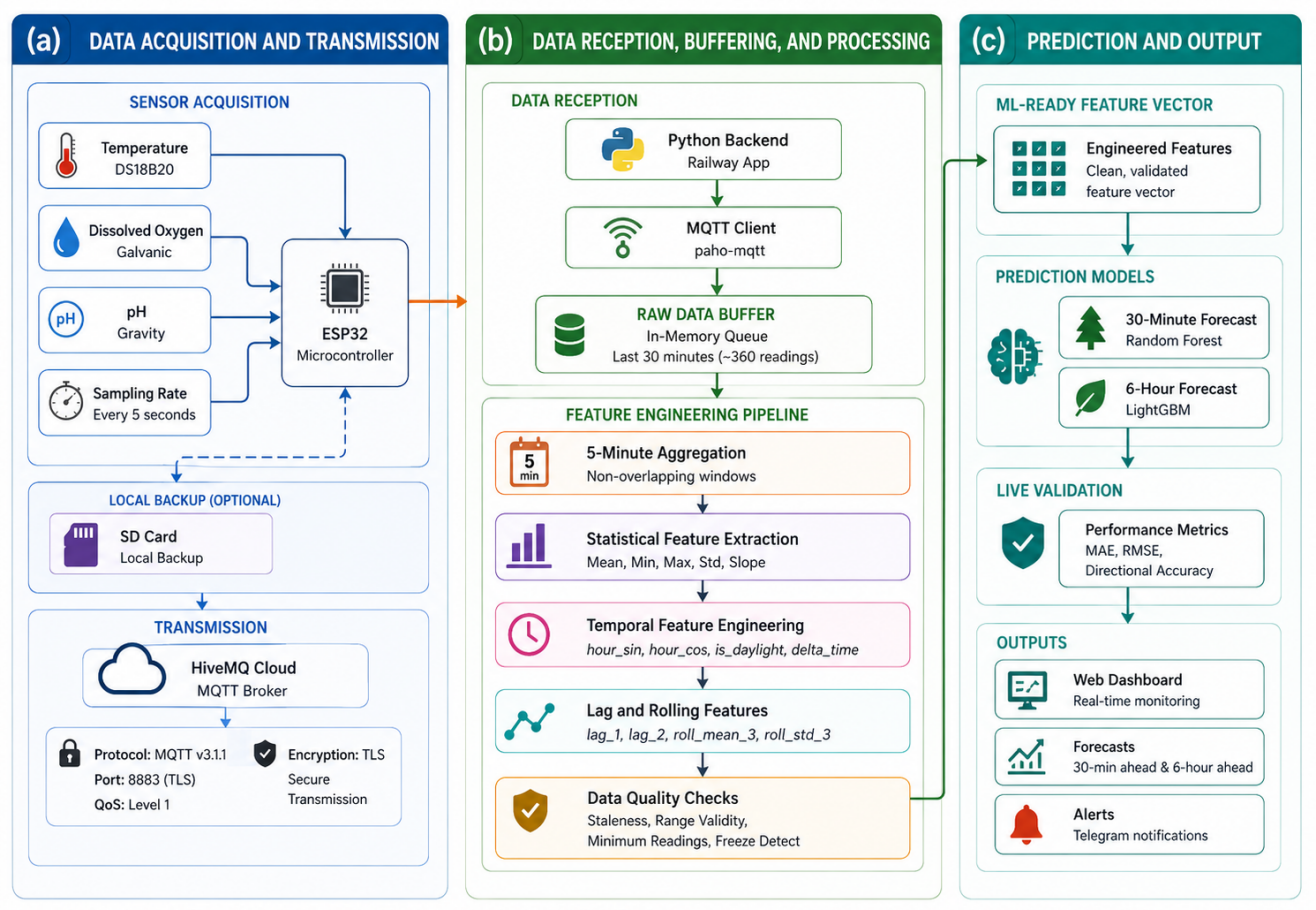
End-to-end workflow: Data acquisition, aggregation, feature construction, offline model selection, and live deployment validation.

This two-phase evaluation design separated offline model selection from deployed-system assessment (Table 6). The live pond validation subset contained 4657 validated 30 min forecasts and 3391 validated 6 h forecasts. The dashboard provided operators with real-time readings, forecast displays, threshold-based alerts, and a qualitative ammonia-risk indicator derived from temperature and pH. The end-to-end workflow from data acquisition through live validation is illustrated in Figure 3.

After the initial 1–10 April live validation period, the same deployed monitoring system continued operation from 1 May to 16 June 2026. This extended field deployment was analysed as additional field-deployment evidence using the same deployed sensing node and the same previously trained Random Forest and LightGBM models. The extended dataset was used to document continued field operation, low-DO event capture, power-outage periods, and forecast behaviour under a longer monitored period, but it was not treated as independent multi-site validation.

## 3. Results

### 3.1. Dataset Preparation and Temporal Split

After feature generation and forecast-target alignment, the processed dataset contained 2034 samples represented as 5 min aggregated windows. Each sample included water-quality statistics (dissolved oxygen, temperature, pH), lag features, rolling statistics, cyclic time-of-day encoding, and operational event timing variables.

A chronological train–validation–test split was applied to preserve temporal order and prevent data leakage. The training set contained 1525 samples (25–30 March 2026; approximately 5.5 days), the validation set contained 305 samples (30–31 March 2026; approximately 1.3 days), and the test set contained 204 samples (31 March to 1 April 2026; approximately 17 h spanning two calendar dates). All three subsets included both daytime and nighttime periods, capturing the diel oxygen cycle. This temporal splitting strategy ensures that the model is evaluated on future data relative to training, simulating real deployment conditions where forecasts must generalise to unseen time periods rather than interpolate within previously observed patterns. The relatively short test-set duration represents a held-out chronological segment; broader field variability is described through the subsequent extended field deployment analysis.

### 3.2. Offline Model Selection and Test-Set Forecasting Behaviour

Seven candidate models were evaluated during offline model development: Linear Regression, Ridge Regression, Support Vector Regression, Random Forest, XGBoost, LightGBM, and ensemble averaging. Model selection was based on validation-set performance, with final evaluation on the held-out test set. The complete candidate model comparison is shown in Table 7.

For the 30 min forecast horizon, Random Forest was selected because it provided the strongest overall validation balance, with the highest $R^{2}$ (0.440) and directional accuracy (55.3%) among the candidates, and validation MAE (0.712 mg/L) close to the best-performing models. On the test set, it achieved an MAE of 0.652 mg/L, $R^{2}$ of −0.069, and a directional accuracy of 51.2%. For the 6 h horizon, LightGBM was selected because it achieved the lowest validation MAE (0.856 mg/L) and highest $R^{2}$ (0.139) among the evaluated candidates; the test MAE was 0.688 mg/L, $R^{2}$ of −2.108, and directional accuracy was 41.9%. The selected model performance is summarised in Table 8. The negative test-set $R^{2}$ values indicate limited explanatory fit on the held-out chronological test period, particularly for the 6 h horizon, and are therefore interpreted together with MAE and directional accuracy rather than as standalone indicators of predictive performance.

**Figure 4:**
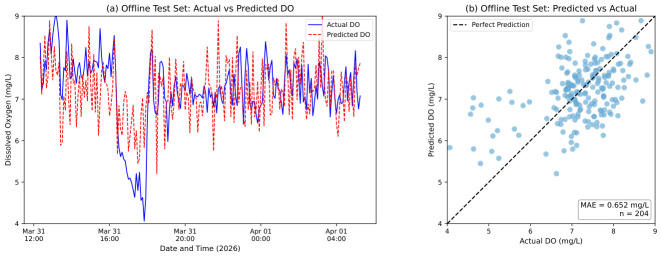
Test-set forecasting behaviour of the 30 min Random Forest model (31 March 12:20 to 1 April 05:15, 2026; approximately 17 h). (**a**) Time-series comparison of actual (blue) and predicted (red dashed) dissolved oxygen values with date/time x-axis. (**b**) Scatter plot of predicted versus actual values with identity line; MAE = 0.652 mg/L, *n* = 204.

The test-set forecasting behaviour of the 30 min Random Forest model is shown in Figure 4. Panel (a) displays the actual and predicted dissolved oxygen values over the test period, while panel (b) shows the corresponding scatter plot with the identity line. The model tracked dissolved oxygen fluctuations during stable periods but showed increased deviation during rapid changes associated with operational events. The MAE value of 0.652 mg/L on the test set represents the offline baseline before cloud-connected deployment.

### 3.3. Live Deployment Validation

After offline model selection, the selected models were integrated into the Railway-hosted backend and evaluated during cloud-connected pond operation from 1 to 10 April 2026. During this period, forecasts were generated continuously from pond sensor data, stored in the forecast log, and later compared with actual dissolved oxygen values observed after each horizon elapsed.

The live pond validation produced 4657 validated 30 min forecasts and 3391 validated 6 h forecasts. The 30 min forecast achieved an MAE of 0.783 mg/L, an RMSE of 1.004 mg/L, and a directional accuracy of 60.23%. The 6 h forecast achieved an MAE of 1.109 mg/L, an RMSE of 1.358 mg/L, and a directional accuracy of 53.82%. An MAE below 1 mg/L for the 30 min horizon suggests that the system may provide trend-awareness information during deteriorating DO conditions, though direct measured-DO alerts remain necessary for threshold-based intervention. These results represent operational performance under real pond sensing conditions, cloud communication, and delayed validation logic. The live deployment validation metrics are summarised in Table 9.

The live $R^{2}$ values were 0.18 for the 30 min horizon and −0.52 for the 6 h horizon, showing modest explanatory fit at the short horizon and limited explanatory fit at the extended horizon. In relative terms, the MAPE was 10.3% for the 30 min horizon and 15.5% for the 6 h horizon, indicating that prediction errors represented approximately one-tenth to one-sixth of observed DO concentrations during live validation. Residual analysis showed a positive mean bias of +0.16 mg/L for the 30 min horizon and +0.76 mg/L for the 6 h horizon, indicating systematic overprediction, especially at the extended horizon.

The models were also compared with a persistence baseline that predicts future DO as equal to the current observation. For the 30 min horizon, the Random Forest model provided a modest MAE reduction relative to persistence (0.783 vs. 0.791 mg/L, approximately 1.0%). For the 6 h horizon, LightGBM showed a higher MAE than persistence (1.109 vs. 0.952 mg/L), indicating limited predictive value at the extended horizon.

The predicted values occupied a compressed range relative to observed DO values: approximately 30% of the observed range for the 30 min horizon and 12% for the 6 h horizon. For the 6 h model, 99.5% of predictions fell within the 8–9 mg/L band.

**Figure 5:**
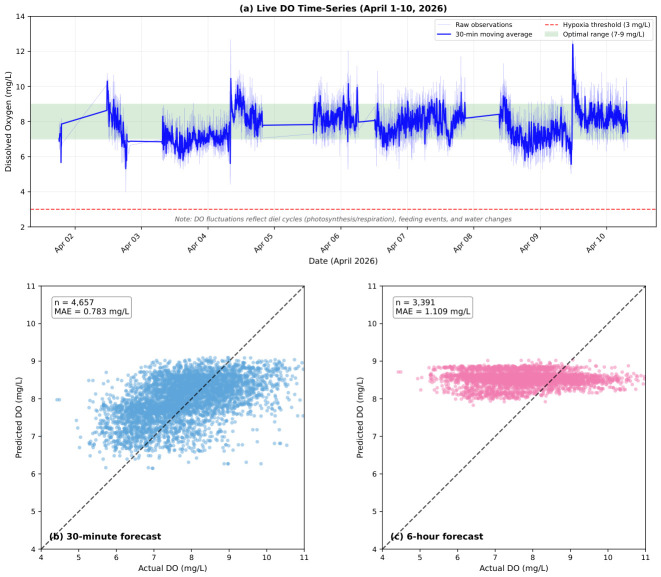
Live deployment validation of dissolved oxygen forecasts (1–10 April 2026). (**a**) Observed dissolved oxygen time-series during the live validation period with 30 min moving average overlay; the horizontal dashed line indicates the 3 mg/L hypoxia threshold; the shaded band indicates the 7–9 mg/L favourable range; one short sensor-anomaly episode containing 0.0 mg/L readings was excluded; DO fluctuations reflect diel cycles, feeding events, and water changes. (**b**) 30 min forecast: Predicted versus actual values (*n* = 4657, MAE = 0.783 mg/L, $R^{2}$ = 0.18). (**c**) 6 h forecast: Predicted versus actual values (*n* = 3391, MAE = 1.109 mg/L, $R^{2}$ = −0.52). Identity lines shown for reference.

The live forecasting behaviour is illustrated in Figure 5. Both horizons tracked dissolved oxygen fluctuations to varying degrees, with the 30 min forecast demonstrating tighter clustering around the identity line than the 6 h forecast. The wider scatter for the extended horizon reflects the increased uncertainty associated with longer prediction windows.

### 3.4. Forecast Horizon Comparison

A direct comparison of the two forecast horizons during live deployment validation revealed consistent performance differences. Prediction error increased with horizon length: MAE rose from 0.783 mg/L (30 min) to 1.109 mg/L (6 h), representing a 41.6% increase. RMSE increased from 1.004 mg/L to 1.358 mg/L, a 35.3% increase. Directional accuracy decreased from 60.23% (30 min) to 53.82% (6 h), indicating reduced ability to predict the direction of dissolved oxygen change at longer horizons.

The horizon comparison is summarised in Table 10. These results indicate that the 30 min forecast provides lower-error short-term prediction, while the 6 h forecast offers broader but less precise planning information.

### 3.5. Feature Importance and Operational Event Effects

Built-in tree-based feature importance ranking showed distinct predictor patterns between the two forecast horizons. For the 30 min Random Forest model, recent dissolved oxygen measurements ranked highest overall, although hours since water change also appeared among the leading predictors. The 15 min rolling mean of DO contributed 26.5% of model importance, followed by hours since water change and several DO-derived statistics, including DO minimum (14.2%), DO maximum (11.8%), and DO mean (9.3%). In the built-in feature-importance ranking, DO-derived features accounted for over 60% of total importance, suggesting that the fitted 30 min model relied heavily on recent oxygen-state variables.

For the 6 h LightGBM model, operational timing variables ranked highest. Hours since water change contributed 29.1% of importance, hours since feeding contributed 18.2%, and hour-of-day contributed 12.4%. Recent DO features remained relevant but contributed less than 25% combined. This shift in built-in model importance suggests that the fitted 6 h model used operational context more strongly than the 30 min model.

Operational event analysis quantified the dissolved oxygen response to farm-management actions. Water-change events were followed by DO recovery, averaging 1.33 mg/L within approximately one hour of the event. The mean DO in the 30 min before water change was 6.82 mg/L, rising to 8.15 mg/L in the 30 min after the event. Feeding events showed the opposite pattern: DO declined by 0.5–1.0 mg/L over two to four hours following feeding, consistent with increased biological oxygen demand from fish metabolism and uneaten feed decomposition.

**Figure 6:**
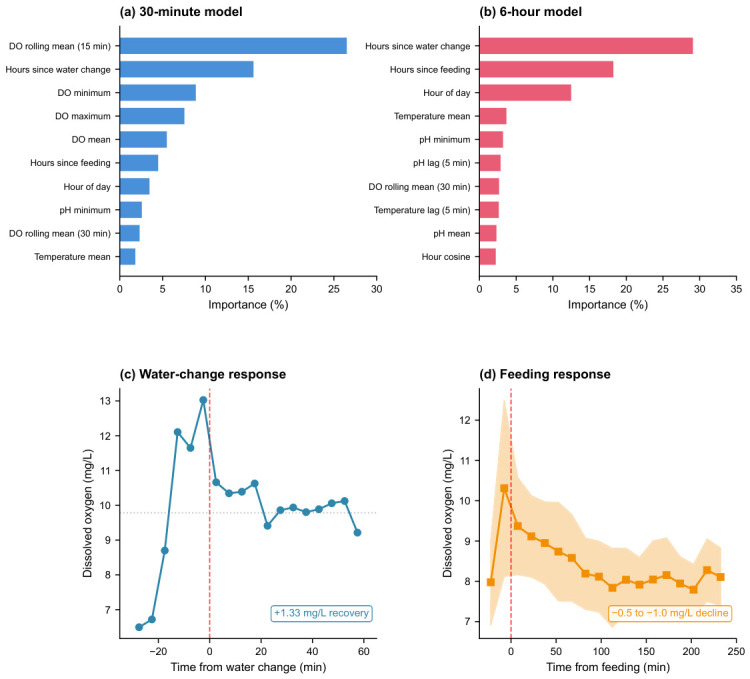
Feature importance and operational event effects on dissolved oxygen. (**a**) Top 10 features for the 30 min Random Forest model, dominated by recent DO statistics. (**b**) Top 10 features for the 6 h LightGBM model, dominated by operational timing variables. (**c**) Dissolved oxygen response around water-change events showing recovery of 1.33 mg/L within one hour; the shaded area indicates ±1 standard deviation across events. (**d**) Dissolved oxygen response around feeding events showing a gradual decline of 0.5–1.0 mg/L over two to four hours; the shaded area indicates ±1 standard deviation across events.

The feature importance distributions and operational event responses are shown in Figure 6. Panel (a) displays the top features for the 30 min model, panel (b) displays the top features for the 6 h model, panel (c) shows the DO time-course around water-change events, and panel (d) shows the DO time-course around feeding events.

### 3.6. Deployment Stability and Data Continuity

The deployed prototype maintained continuous operation throughout the live pond validation period (1–10 April 2026). The system recorded 102,670 raw sensor readings at approximately 5 s intervals, generated 3041 aggregated 5 min windows, produced 4657 validated 30 min forecasts, and produced 3391 validated 6 h forecasts. Raw readings, aggregated records, forecasts, and validation logs were retained through persistent CSV-based storage on the Railway-hosted backend.

Initial deployment revealed measurement stability challenges typical of outdoor pond environments. During the first deployment days, anomalous readings occurred, including occasional zero dissolved oxygen values, voltage instability, and elevated pH readings associated with direct sunlight exposure on the sensor housing. These issues were addressed through improved power regulation, cable shielding, and repositioning of the sensor casing to reduce direct sun exposure. After these adjustments, the system operated continuously throughout the remainder of the validation period with reduced anomaly rates compared to the initial deployment. The deployment continuity and stability metrics are summarised in Table 11.

### 3.7. Extended Field Deployment

Following the initial validation, the forecasting system was deployed continuously for an extended 47-day period (1 May–16 June 2026) to document continued field operation under real pond conditions. During this deployment, the system recorded 624,706 sensor readings at approximately 6.5 s intervals; of these, 620,276 readings (99.3%) contained valid DO measurements. Two power outage events (12 May and 10 June) caused sensor downtime totalling 8 h, during which the DO probe was offline, and valid DO measurements could not be obtained. Four low-DO events were captured by the deployed sensor, with minimum readings of 1.50, 1.88, 2.29, and 2.66 mg/L; event causes (aerator malfunction, overcrowding stress, pipe disconnection, and overfeeding) were documented by the system operator (Table 12). Two additional events during power outages required operator response based on situational awareness and farm observation rather than continuous sensor measurements.

Over the extended deployment period, the forecasting models, which were trained on data from late March 2026, achieved a mean absolute error (MAE) of 0.46 mg/L for 30 min predictions and 0.75 mg/L for 6 h predictions, with $R^{2}$ values of 0.749 and 0.076, respectively. Compared to a persistence baseline (predicting that future DO equals the most recent observation), the 30 min model improved the MAE by 19.6% and the 6 h model by 52.0%, indicating better-than-persistence performance during this monitored period. The direction accuracy (correctly predicting whether DO would increase or decrease) was 53.0% for 30 min and 87.2% for 6 h forecasts. Because the records were collected from a single pond and the consecutive high-frequency measurements are temporally correlated, these extended results should be interpreted as additional field-deployment evidence rather than independent validation of generalisable forecasting performance.

**Figure 7:**
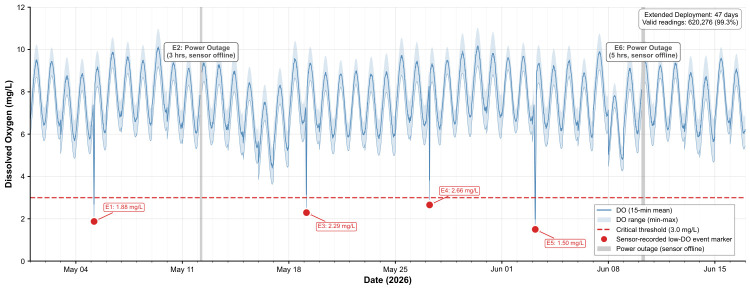
Extended field deployment timeline from 1 May to 16 June 2026. The line shows dissolved oxygen trends from valid sensor readings. Red markers indicate four sensor-recorded low-DO events, while the grey-shaded regions indicate two documented power-outage periods, during which the sensing node was offline and valid DO measurements could not be obtained. These data provide additional field-deployment evidence but do not remove the limitations of single-pond deployment, temporal correlation, and the lack of independent reference-meter validation.

The extended deployment timeline is presented in Figure 7, showing DO trends from valid sensor readings with sensor-recorded low-DO event markers and power outage periods. Summary statistics are provided in Table 13.

## 4. Discussion

### 4.1. Forecasting Performance and Error Characteristics

Live field evaluation confirmed that short-horizon forecasting provides lower-error trend-awareness information than extended-horizon forecasting for dissolved oxygen management. The 30 min model achieved a substantially lower error than the 6 h model (approximately 30% lower MAE), with higher directional accuracy. This performance difference reflects fundamental characteristics of dissolved oxygen dynamics in aquaculture ponds. At short horizons, dissolved oxygen changes are largely deterministic: if oxygen is declining due to respiration, it will likely continue declining in the near term unless an intervention occurs. The strong autocorrelation of dissolved oxygen over periods of 15–30 min allows models to extrapolate the recent trajectory effectively [9]. At longer horizons, however, multiple stochastic factors accumulate: diel photosynthesis cycles shift oxygen production and consumption, feeding events increase biological oxygen demand, temperature fluctuations alter oxygen solubility, and unrecorded disturbances (cloud cover, fish activity, and partial water exchange) introduce variability that recent measurements cannot fully capture. The feature-importance shift observed between horizons supports this interpretation: 30 min predictions relied primarily on recent dissolved oxygen values, whereas 6 h predictions depended more heavily on operational timing variables that capture the broader contextual state.

The persistence comparison indicates that short-horizon DO prediction is strongly influenced by temporal autocorrelation. The 30 min model provided only a modest improvement over persistence, while the 6 h model underperformed the persistence baseline. Therefore, the extended-horizon forecast should be interpreted as a trend-support indicator rather than a standalone decision trigger. The systematic positive bias suggests regression-to-mean behaviour, where predictions gravitated toward the central DO range. This behaviour reduces sensitivity to low-DO excursions and should be considered when interpreting forecast outputs.

These results warrant critical interpretation. During the April live validation, the 30 min Random Forest model improved the MAE over persistence by only approximately 1% (0.783 vs. 0.791 mg/L), indicating that most of the predictable signal at this horizon was already contained in recent DO history rather than in additional learned relationships beyond recent-state features. The 6 h LightGBM model performed worse than persistence (MAE 1.109 vs. 0.952 mg/L), exhibited negative live $R^{2}$ (−0.52), positive bias (+0.76 mg/L), and severe prediction-range compression (99.5% of predictions within 8–9 mg/L). This compression behaviour, where predictions gravitated toward central DO values rather than tracking actual fluctuations, was an observed empirical pattern in this deployment and limits the model’s ability to anticipate low-DO threshold excursions. The 6 h forecast should, therefore, not be treated as a standalone threshold-event prediction tool; measured-DO alerts remain essential for hypoxia detection [9]. The extended 47-day field deployment strengthens the engineering and field-evaluation contribution by documenting continued field operation, sensor-recorded low-DO events, and power-outage challenges, but it does not remove the fundamental limitations of single-pond data, temporally correlated high-frequency measurements, and the absence of independent reference-meter validation [12]. Accordingly, this study should be interpreted as a proof-of-concept field assessment of a low-cost monitoring-and-forecasting workflow rather than as validation of an operational early-warning system.

From an operational perspective, 30 min trend-awareness information may be operationally relevant. It may allow time for operators to inspect the system, increase aeration rate, reduce or delay feeding, or prepare corrective intervention before conditions deteriorate further. The 6 h forecast, while less precise, may serve as a planning indicator. It could potentially inform shift scheduling, feed timing, or anticipation of overnight oxygen decline, though the limitations described above apply. The distinction between short-term trend-awareness information and longer-term planning indicators aligns with practical aquaculture workflows where immediate response and longer-term preparation require different information granularity [29,30].

### 4.2. Live Field Evaluation

Separating offline model selection from live deployment validation was central to this study’s methodology. Offline evaluation using chronological train–validation–test splits was necessary for selecting Random Forest (30 min) and LightGBM (6 h) from candidate algorithms, but offline metrics alone do not capture operational behaviour. The live validation stage evaluated forecasts generated during continuous system operation, stored alongside prediction timestamps, and later matched with observed dissolved oxygen values recorded at the corresponding target times.

This distinction is important because models that perform well on held-out test sets may behave differently when exposed to real-time data streams, changing sensor conditions, communication latency, and deployment-related noise. Many smart aquaculture studies demonstrate either monitoring architectures or offline prediction accuracy, but fewer studies evaluate forecasting behaviour within continuous operational pipelines [11,31]. By recording and matching predictions during live pond operation, this study provides a field-based assessment of forecasting behaviour rather than relying on offline benchmarking alone. The live validation sample sizes (*n* = 4657 and *n* = 3391) provided an initial description of error distributions and directional accuracy during the April 2026 pond deployment period. However, because consecutive 5 min windows are temporally correlated and forecasts overlap in time, the effective number of independent observations is substantially smaller than the nominal forecast count, and the reported error distributions should be interpreted accordingly. The variation in $R^{2}$ across evaluation stages—from validation (0.440 for 30 min, 0.139 for 6 h) to test (−0.069, −2.108) and to April live deployment (0.18, −0.52)—suggests that validation-set performance was optimistic relative to chronologically harder periods, and that generalisation to unseen time windows remains limited for both horizons. The extended May–June deployment showed improved $R^{2}$ values (0.749 for 30 min, 0.076 for 6 h). Analysis of the extended deployment predictions revealed that the severe prediction-range compression observed in April (where 99.5% of 6 h forecasts fell within the 8–9 mg/L band) was substantially reduced, with prediction spread covering approximately 104% of the actual DO range. This suggests that over the longer 47-day continuous deployment, the models—particularly at the 30 min horizon—were better able to track DO fluctuations rather than regressing toward central values. However, the improvement should be interpreted cautiously: the longer continuous series may allow lag-based features to exploit stronger temporal autocorrelation, and single-pond deployment with temporally correlated high-frequency data remains a fundamental limitation that prevents attribution of the improved $R^{2}$ to genuine model generalisation.

### 4.3. Operational Event Effects

Feature importance analysis revealed that dominant predictors differed systematically between forecast horizons. For 30 min prediction, recent dissolved oxygen measurements provided the strongest signal, particularly rolling means over 15 and 30 min windows. The model effectively extrapolated the current trajectory, relying on measurement recency rather than contextual variables. For 6 h prediction, operational timing features dominated: hours since water change, hours since feeding, and hour-of-day collectively contributed over 60% of model importance. This shift indicates that extended-horizon forecasting requires contextual information about system state and management history, not only immediate sensor values.

The use of continuous time-since-event features rather than binary event flags proved important. A binary indicator marks only whether an event occurred, whereas elapsed time captures the gradual nature of dissolved oxygen response. Feeding increases biological oxygen demand through fish metabolism, uneaten feed decomposition, and microbial activity. These effects develop over hours rather than minutes [7]. Water exchange, by contrast, can improve oxygen saturation more rapidly, with recovery effects observable within 30–60 min. Event-response analysis confirmed these dynamics: water changes produced a mean recovery of approximately 1.33 mg/L within 60 min, while feeding induced a gradual decline of 0.5–1.0 mg/L over several hours. These findings suggest that dissolved oxygen forecasting in intensive aquaculture should not be treated as a purely sensor-driven regression problem; structured recording of farm-management events strengthens predictive modelling for horizons beyond very short windows.

The feature-importance values reported here are built-in tree-based model metrics (Gini importance for Random Forest and split-gain importance for LightGBM) and should, therefore, be interpreted as model-specific associations rather than causal evidence. These measures indicate which variables contributed most strongly to the fitted predictions, but they do not prove that operational events directly caused the observed dissolved oxygen changes under controlled conditions. Additionally, correlated predictors can affect tree-based importance rankings, potentially inflating importance for variables that share information with other features. Complementary interpretability methods such as permutation importance or SHAP could help disentangle these effects and provide additional insight in future work. Nevertheless, the observed importance of feeding, water-change timing, and time-of-day is consistent with known aquaculture oxygen dynamics and supports their inclusion as operational context features.

### 4.4. Sensor and Deployment Constraints

Deployment conditions strongly influenced sensing reliability, and iterative adjustment proved valuable for maintaining continuous pond operation. The initial pond deployment exposed the system to environmental and electrical interference that produced anomalous readings, including zero dissolved oxygen values, voltage fluctuations, and pH measurements affected by direct sunlight on the sensor housing. These challenges are consistent with previous reports of field instability in low-cost aquaculture monitoring systems [18,32].

Practical adjustments addressed these issues progressively. Power regulation was improved through voltage stabilisation, cable management was enhanced with shielding, and sensor positioning was adjusted to reduce direct sun exposure. After these modifications, the system operated continuously throughout the validation period, with anomaly rates declining substantially. This experience suggests that low-cost IoT prototypes may require iterative field adjustment rather than laboratory-only development. Model performance cannot be evaluated separately from sensing quality; a forecasting pipeline is only as reliable as its input data stream. The pond deployment demonstrated that continuous operation was observed with commodity hardware in this deployment when practical deployment constraints were addressed systematically.

### 4.5. Comparison with Prior Studies

This study occupies a position between two common directions in smart aquaculture research: monitoring-focused IoT systems and algorithm-focused dissolved oxygen prediction. Monitoring systems demonstrate the feasibility of collecting and displaying water-quality data but typically provide limited evidence of predictive forecasting under field conditions [31,33]. Algorithm-focused studies report strong prediction performance using historical datasets but do not always demonstrate how models operate when connected to live sensing, real-time feature generation, and continuous validation [16,34]. In contrast, this study integrates low-cost sensing, cloud-based processing, calibration procedures, feature engineering, multi-horizon forecasting, event logging, live validation, and dashboard-based monitoring within a single workflow. Table 14 summarises representative dissolved oxygen forecasting and smart aquaculture monitoring studies and positions the present work relative to prior approaches.

Compared with recent dissolved oxygen forecasting studies that report lower errors and higher explanatory fit under different sensing and validation conditions, the present live deployment produced a higher MAE and a lower $R^{2}$. For example, Feng et al. achieved $R^{2}=0.997$ using a wavelet-denoising and optimised SVR approach with professional-grade sensors [34]. However, direct numerical comparison is limited because prior studies differ in species, water system type, sensor quality, forecast horizon, data duration, and whether forecasts were validated during continuous field operation rather than on held-out historical data. Some comparison studies focus on monitoring rather than forecasting [18,32], while others report prediction accuracy without live deployment validation [16,35]. The value of the present study, therefore, lies not in algorithmic superiority but in providing a preliminary field benchmark for low-cost dissolved oxygen forecasting in an outdoor Nile tilapia pond, with event-aware features and transparent reporting of performance limitations.

The field-evaluation workflow demonstrated here represents a methodological structure that may be considered for adaptation in other low-cost aquaculture forecasting studies. The workflow comprises five linked components: (1) calibrated low-cost sensing with documented accuracy bounds, (2) cloud-based data aggregation and feature engineering, (3) operational event logging that captures time-since-feeding and time-since-water-change as continuous predictors, (4) stored forecasts with prediction timestamps, and (5) delayed validation by matching predictions against subsequently observed values under continuous field operation. This workflow provides a structure for preliminary field evaluation of forecasting behaviour under realistic deployment constraints rather than relying solely on held-out test sets from historical data. The workflow is hardware-agnostic and can be implemented with different sensor configurations or cloud platforms, which could be considered for adaptation in other contexts pending further validation.

The contribution should be understood as an integrated monitoring-and-forecasting prototype rather than a novel forecasting algorithm. Random Forest and LightGBM are established methods; their value in this context lies in practical embedding within a low-cost IoT architecture designed for Nile tilapia aquaculture. Oman’s aquaculture sector faces challenges characteristic of warm-climate, water-limited environments: high ambient temperatures accelerate oxygen depletion, and smaller-scale operations may lack resources for commercial monitoring infrastructure [36]. Professional aquaculture monitoring systems generally offer stronger measurement stability, manufacturer support, and long-term operational robustness, but their capital and maintenance requirements may limit uptake in smaller-scale or resource-constrained settings [12]. A low-cost system that provides 30 min trend-awareness information and interpretable 6 h planning indicators may address a practical gap by providing operator-facing monitoring without overclaiming autonomous control capability [29]. The prototype suggests that commodity hardware can support preliminary dissolved oxygen monitoring and short-horizon forecasting, but the results remain context-specific and require stronger validation before operational use.

### 4.6. Extended Deployment Observations

The extended 47-day field deployment provides additional evidence of continued system operation under real-world conditions, including four sensor-recorded low-DO events and two power outage periods that reflect the practical challenges of aquaculture IoT systems. The forecasting models produced valid forecasts during the extended deployment period, achieving a 30 min MAE of 0.46 mg/L and a 6 h MAE of 0.75 mg/L. The two power outage events highlight an important field limitation of the system: while the sensor was offline during these critical periods (totalling 8 h), valid DO measurements could not be obtained, and operator response was based on situational awareness and farm observation rather than continuous sensor measurements. This underscores that the IoT system served as one component of a broader farm management strategy rather than an autonomous decision system. The extended deployment improves the practical relevance of the study, but it does not remove the need for multi-pond, multi-season, independently reference-validated evaluation before operational use. Future work should address sensor redundancy and backup power systems to minimise data loss during power outages, which represent a significant risk factor in resource-limited aquaculture settings.

## 5. Limitations

Several limitations should be acknowledged. The study was conducted at a single pond site with one monitoring node. Although the extended field deployment (1 May to 16 June 2026) provided 47 days of operation and captured four sensor-recorded low-DO events, this remained a single-pond, single-sensor deployment without multi-site or multi-season coverage. Multi-node deployment with spatial averaging would likely improve measurement representativeness and forecasting robustness.

The models exhibited prediction compression, with predicted values spanning only a limited portion of the observed DO range, particularly for the 6 h horizon. Because the models did not generate predicted DO values below approximately 6 mg/L, prediction-based threshold alerts for hypoxia should not be used alone. Direct real-time alerts based on measured DO remain essential, while model forecasts should be interpreted as trend-awareness support rather than standalone early-warning indicators.

The system relied on low-cost sensors, which introduced sensitivity to calibration stability, electrical noise, and environmental exposure. The SEN0237-A dissolved oxygen sensor has lower accuracy than laboratory-grade instruments. This cost–accuracy trade-off is acceptable for trend monitoring but may limit precision in applications requiring high-accuracy threshold detection [12]. The pH sensor showed reduced reliability during deployment and was treated as a supporting monitoring variable rather than a critical alerting input.

The deployed dissolved oxygen sensor provided both the input features and the future target values used for forecast validation. Therefore, the validation evaluates forecasting consistency against future readings from the calibrated deployed sensor, rather than independent absolute dissolved oxygen accuracy against a reference instrument. The sensor-recorded low-DO events during the extended deployment were not independently verified using a reference DO meter. Future deployments should include periodic reference-meter checks to quantify measurement accuracy.

The prototype was operated using a wired pond-side power supply. The two power outage events during the extended deployment (totalling 8 h) resulted in sensor downtime where valid DO measurements could not be obtained. Future deployments should address sensor redundancy and backup power systems to minimise data loss during power interruptions.

Dynamic temperature compensation was not implemented in the DO conversion. Deployments spanning wider thermal ranges or seasonal variation should incorporate formal temperature-compensated DO correction and periodic recalibration.

The ammonia-risk component was implemented as a rule-based indicator rather than a direct ammonia measurement. Direct ammonia sensing or laboratory water-quality testing would be needed to validate ammonia-related risk more rigorously.

Additionally, the study focused on water-quality monitoring and dissolved oxygen forecasting rather than on direct biological performance outcomes. Although live Nile tilapia were present in the pond, the study did not include a formal growth-performance trial or controlled welfare assessment.

Finally, the forecasting models were developed and validated using data from one prototype configuration and one local Omani aquaculture context. External validation across additional farms, pond types, seasons, and operational contexts would be necessary before broader application.

## 6. Conclusions and Future Work

This study presents a preliminary field evaluation of a low-cost IoT workflow for dissolved oxygen monitoring and short-horizon forecasting in Nile tilapia pond aquaculture in Oman. The prototype integrated calibrated low-cost sensing, cloud communication, event logging, dashboard visualisation, stored forecast matching, and machine-learning prediction within a single field-deployed workflow.

The April live validation showed that the forecast value was strongly horizon-dependent. The 30 min Random Forest model produced a lower error than the 6 h LightGBM model, but its improvement over persistence was modest, indicating that much of the short-horizon signal was already contained in recent dissolved oxygen history. The 6 h model underperformed persistence and exhibited prediction-range compression, limiting its suitability for threshold-event forecasting. These findings show that, in the present configuration, model outputs are better interpreted as supplementary trend-awareness information rather than as standalone early-warning indicators.

The extended 47-day deployment provided additional field evidence by documenting continued operation, four sensor-recorded low-DO events, and two power-outage periods. However, the study remains limited by single-pond deployment, temporally correlated high-frequency measurements, low-cost sensor constraints, and the absence of independent reference-meter validation. Future work should, therefore, prioritise multi-pond and multi-season evaluation, reference-instrument verification, sensor redundancy, backup power, formal temperature compensation, and integration with biological performance outcomes.

Overall, the contribution of this work is not the validation of a generalisable operational forecasting system, but a transparent proof-of-concept field assessment showing both the potential and the practical limits of low-cost IoT-based dissolved oxygen forecasting in small-scale tilapia aquaculture.

## Figures and Tables

**Table 1:** Hardware and sensing components.

Component	Function	Parameter	Interface	Operational Notes
ESP32 DevKit V1	Processing and communication	—	Wi-Fi, GPIO, ADC	Dual-core MCU; handles sensing, MQTT, cloud sync
Galvanic DO Probe	DO measurement	DO (mg/L)	Analogue	Air saturation calibration; requires stable power
DS18B20	Temperature measurement	Temp (°C)	Digital (1-Wire)	Waterproof; three-point calibration applied
Gravity pH Sensor V2	pH measurement	pH	Analogue	Two-point buffer calibration (pH 4, pH 7)
Air pump with diffusers	Aeration	—	—	Maintains suitable DO for live tilapia
Protective enclosure	Electronics protection	—	—	Waterproof housing for ESP32 and boards

**Table 2:** Sensor calibration summary.

Sensor	Method	Calibration Points	Final Equation	Verification	Notes
DS18B20	Three-point linear regression	2.70/1.3; 20.82/21.8; 36.75/38.3 °C	$T_{c}=1.088T_{r}-1.386$	±0.3 °C	Acceptable for prediction
DO Probe	Two-point scaling	Zero-O_2_: 0 V; Air-sat: 0.628 V	DO=13.648·V	8.3–8.6 mg/L	NaOH electrolyte added
pH Sensor	Two-point buffer	pH 7: 1.398 V; pH 4: 1.912 V	pH=−5.839V+15.16	±0.05 units	Drift-sensitive

**Table 3:** Dataset acquisition and storage summary.

Data Source	Date Range	Sampling	Records	Purpose
Stage 1: Offline Model Development				
Training set	March 2026	5 min windows	1525	Model training
Validation set	March 2026	5 min windows	305	Model selection
Test set	March–April 2026	5 min windows	204	Offline evaluation
Stage 2: Live Deployment Validation				
Raw sensor readings	1–10 April 2026	5 s intervals	102,670	Real-time monitoring
Aggregated windows	1–10 April 2026	5 min windows	3041	Feature generation
Forecast validation log	1–10 April 2026	Per-forecast record	4657 (30 min); 3391 (6-h)	Live accuracy evaluation

**Table 4:** Feature groups used for dissolved oxygen prediction.

Feature Group	Example Features	Purpose	30 min	6 h
Water-quality statistics	DO mean, min, max, std; Temp mean, std; pH mean	Current state and variability	✓	✓
Short-term change	DO change from previous window; Temp change	Immediate trend direction	✓	✓
Lag features	DO at t−1, t−2, t−3; Temperature at t−1	Recent DO and environmental history	✓	✓
Rolling statistics	Rolling mean (30 min, 1 h); Rolling std	Recent trend and stability	✓	✓
Time-of-day	Hour (cyclic sine/cosine encoding)	Daily patterns	✓	✓
Operational events	Time since feeding; Time since water change	Farm-management effects	✓	✓

**Table 5:** Candidate models considered during offline development.

Model	Model Type	Role in Comparison	Final Use
Linear Regression	Linear baseline	Simple baseline	Not selected
Ridge Regression	Regularised linear	Regularised baseline	Not selected
Support Vector Regression	Kernel-based	Non-linear kernel approach	Not selected
Random Forest	Tree-based ensemble	Non-linear ensemble	Selected for 30 min
XGBoost	Gradient boosting	Gradient-boosted trees	Not selected
LightGBM	Gradient boosting	Efficient gradient boosting	Selected for 6 h
Ensemble (averaging)	Model averaging	Combined prediction	Not selected

**Table 6:** Two-stage evaluation design.

Stage	Date Range	Purpose	Records	Output
Stage 1: Offline Model Development				
Training set	March 2026	Fit candidate models	1525 samples	—
Validation set	March 2026	Model comparison	305 samples	Model selection
Test set	March–April 2026	Final offline evaluation	204 samples	Offline metrics
Stage 2: Live Deployment Validation				
30 min forecasts	1–10 April 2026	Short-horizon validation	4657 validated	Live metrics
6 h forecasts	1–10 April 2026	Extended-horizon validation	3391 validated	Live metrics

**Table 7:** Candidate model comparison during offline development.

Horizon	Model	Val MAE (mg/L)	Val RMSE (mg/L)	Val $R^{2}$	Val Dir. (%)
30 min forecast					
	Linear Regression	0.813	0.945	0.373	51.3
	Ridge Regression	0.795	0.929	0.395	51.6
	SVR (RBF)	0.868	1.144	0.083	47.4
	**Random Forest**	**0.712**	**0.893**	**0.440**	**55.3**
	XGBoost	0.706	0.930	0.393	53.6
	LightGBM	0.722	0.907	0.423	49.0
	Ensemble (Avg)	0.708	0.897	0.436	53.6
6 h forecast					
	Linear Regression	2.693	2.937	−4.22	52.0
	Ridge Regression	2.672	2.915	−4.14	53.0
	SVR (RBF)	1.095	1.367	−0.13	54.3
	Random Forest	0.969	1.238	0.073	50.0
	XGBoost	0.903	1.250	0.055	50.0
	**LightGBM**	**0.856**	**1.194**	**0.139**	**50.7**

Note: Model selection was based on validation-set performance. Test-set metrics are reported for selected models in Table 8. Bold indicates the selected model for each horizon. Ensemble averaging was evaluated only for the 30 min horizon.

**Table 8:** Offline model-selection performance for the selected dissolved oxygen forecasting models.

Horizon	Model	Set	MAE (mg/L)	$R^{2}$	Dir. Acc. (%)
30 min	Random Forest	Validation	0.712	0.440	55.3
		Test	0.652	−0.069	51.2
6 h	LightGBM	Validation	0.856	0.139	50.7
		Test	0.688	−2.108	41.9

**Table 9:** Live deployment validation performance of the dissolved oxygen forecasting models from 1 April to 10 April 2026.

Forecast Horizon	Validated Forecasts	MAE (mg/L)	RMSE (mg/L)	$R^{2}$	Direction Accuracy
30 min	4657	0.783	1.004	0.18	60.23%
6 h	3391	1.109	1.358	−0.52	53.82%

**Table 10:** Forecast horizon comparison during live deployment validation.

Horizon	Model	*n*	MAE (mg/L)	RMSE (mg/L)	Dir. Acc. (%)
30 min	Random Forest	4657	0.783	1.004	60.23
6 h	LightGBM	3391	1.109	1.358	53.82
Change (30 min to 6 h)		—	+41.6%	+35.3%	−6.41 pp

**Table 11:** Deployment continuity and stability summary for the pond-deployed prototype (1–10 April 2026).

Category	Result	Interpretation
Raw readings	102,670 records	Continuous sensor acquisition during pond operation
Aggregated windows	3041 records	Stable generation of 5 min forecasting inputs
30 min validated forecasts	4657 records	Live short-horizon forecast validation
6 h validated forecasts	3391 records	Live extended-horizon forecast validation
Validation period	10 days	Continuous cloud-connected operation
Data completeness	98.7%	Minimal gaps during the April monitored period
Forecast generation rate	99.2%	Consistent prediction pipeline execution

**Table 12:** Documented low-DO and outage events during extended field deployment.

Event	Date	Cause ^a^	Min DO (mg/L)	Status
1	5 May	Aerator malfunction	1.88	Sensor-recorded
2	12 May	Power outage	N/A ^b^	Sensor offline (3 h)
3	19 May	Overcrowding stress	2.29	Sensor-recorded
4	27 May	Pipe disconnected	2.66	Sensor-recorded
5	3 June	Overfeeding	1.50	Sensor-recorded
6	10 June	Power outage	N/A ^b^	Sensor offline (5 h)

^a^ Causes were documented by the system operator; ^b^ DO values during power outages could not be obtained as the sensor was offline.

**Table 13:** Extended field deployment summary (1 May–16 June 2026).

Metric	Value
Deployment Overview	
Deployment period	1 May–16 June 2026
Duration	47 days
Total sensor readings	624,706
Valid readings (DO > 0)	620,276 (99.3%)
Power outage periods	2 (totalling 8 h)
DO Statistics (valid readings only)	
DO mean (SD)	7.67 (1.30) mg/L
DO range	1.50–11.04 mg/L
Readings below 5.0 mg/L	9405 (1.52%)
Readings below 3.0 mg/L	773 (0.12%)
Documented Events	
Sensor-recorded low-DO events	4
Documented power outages	2 (totalling 8 h)
Forecast Performance	
Valid forecast predictions	26,706
30 min MAE	0.46 mg/L
6 h MAE	0.75 mg/L
30 min $R^{2}$	0.749
6 h $R^{2}$	0.076
30 min improvement over persistence	+19.6%
6 h improvement over persistence	+52.0%

**Table 14:** Comparison with related dissolved oxygen forecasting and smart aquaculture monitoring studies.

Study	Method/Type	Hardware	Validation	Key Contribution
Feng et al. [34]	WTD-GWO-SVR; prediction	Professional	Offline	High-accuracy DO prediction ($R^{2}=0.997$)
Khudoyberdiev et al. [35]	LSTM, fuzzy; control	Not specified	Offline	Predictive optimisation framework
Hridoy et al. [16]	TFN, AEI; prediction	Low-cost IoT	Offline	Multi-parameter modelling
Sitranata et al. [32]	Cloud IoT; monitoring	Low-cost	Offline	Real-time monitoring architecture
Mohd Jais et al. [18]	Calibration; monitoring	Low-cost	Offline	Sensor calibration ($R^{2}=0.92$)
This study	RF, LightGBM; prediction	Low-cost	Live	Preliminary field evaluation; event-aware features; Oman tilapia pond

Notes: WTD-GWO-SVR = Wavelet threshold denoising with grey wolf-optimised SVR; TFN = TensorFlow neural network; AEI = Aqua Enviro Index; RF = Random Forest. This study is distinguished by live outdoor pond deployment in Oman, delayed forecast validation against subsequently observed DO values, and event-aware features (time since feeding, time since water change).

## Data Availability

The datasets supporting the initial April 2026 live validation are publicly available at: https://github.com/AhmedTheNetCoder/DO-Forecasting-Tilapia-Dataset (accessed on 30 June 2026). The extended May–June 2026 field-deployment dataset analysed during the revision stage is available from the corresponding author upon reasonable request. The extended dataset is not currently deposited in the public repository because data collection and cleaning for the longer monitoring campaign are ongoing.

## Abbreviations

The following abbreviations are used in this manuscript:

DO	Dissolved oxygen
IoT	Internet of Things
MQTT	Message Queuing Telemetry Transport
MAE	Mean absolute error
RMSE	Root mean squared error
ESP32	Espressif 32-bit microcontroller
